# Amyloid Goiter in a 57-Year-Old Woman With Chronic Kidney Disease: A Case Report

**DOI:** 10.7759/cureus.66167

**Published:** 2024-08-05

**Authors:** A. Rahman Farahat, Mahera Roohi

**Affiliations:** 1 Department of Pathology, Blood Bank and Laboratory Medicine, King Hamad University Hospital, Manama, BHR

**Keywords:** amyloid deposition, thyroid gland, chronic kidney disease, amyloidosis, amyloid goiter

## Abstract

Amyloid goiter (AG) is a condition in which amyloid protein builds up in the thyroid gland. Patients with such a condition tend to have thyroid tissue that is extensively involved by amyloid; however, patients are usually euthyroid. Systemic amyloidosis is one of the conditions that may cause damage to the kidneys or worsen the condition of kidney failure in patients with ongoing chronic kidney disease as amyloid proteins can deposit in a variety of tissues including kidneys. Thyroid goiter can rarely be the first confirmed place to be involved with amyloidosis. We present the case of a 57-year-old female with AG who had a history of renal failure.

## Introduction

Amyloidosis refers to a variety of conditions in which amyloid proteins are abnormally deposited in organs and/or tissues. Amyloidosis is a rare occurrence in the thyroid gland [1-2]. This deposition of amyloid protein in the thyroid gland is defined as amyloid goiter (AG), a condition that was first presented by Rockitansky in 1855 and named ‘‘amyloid goiter'' by Eiselberg in 1904 [1].

Despite the rare presentation of the condition, it has been considered a well-established entity, and previous literature has tried to link it to other co-occurring thyroid conditions, such as hyperthyroidism, hypothyroidism, and subacute thyroiditis [2]. Other literature has established the relationship between kidney failure and systemic amyloidosis, especially in patients with advanced disease who undergo dialysis, whether hemodialysis or peritoneal dialysis, for a period that exceeds five years. Dialysis-related amyloidosis is the name given for this condition [2], but AG was rarely the first presentation of systemic amyloidosis and dialysis-related amyloidosis.

AG should be suspected in all patients with progressive, rapidly growing bilateral thyroid enlargement with concomitant systemic inflammatory processes or in patients undergoing hemodialysis treatment [3-4]. The diagnosis of this rare entity is usually made postoperatively, with the current gold standard test being a postoperative histopathological examination of the thyroid tissue under the microscope. This case report represents a patient with chronic kidney disease who was also suffering from goiter. Ultimately, the diagnosis revealed thyroid amyloidosis as a first presentation of goiter.

## Case presentation

A 57-year-old female with a known case of chronic kidney disease and the beta-thalassemia trait presented to the ENT outpatient clinic complaining of neck swelling that had been developing over the past two and a half years. She also reported symptoms of hypocalcemia, including paresthesia, muscle spasms, and muscle cramps. At the point of the presentation, the patient was on follow-up with the nephrology team for impaired renal function with creatinine levels of 244 umol/L. The patient had no previous history of thyroid-related illness, autoimmune conditions, or chronic inflammation, and her family history was negative for any thyroid-related or systemic inflammatory conditions, including autoimmune diseases. The patient had no history of dialysis at the time of presentation.

Initially, due to the COVID-19 pandemic, teleconsultation was done instead of a physical examination. This condition was addressed later in the next visit during the examination, and bilateral swelling in the neck region was observed, with the left side being more prominent. The patient was confirmed to have goiter on physical examination, and thus, lab investigations along with an ultrasound were requested.

Laboratory investigations revealed a slight uptake in parathyroid hormone (PTH) levels (109.7), but calcium remained within the normal range (2.26), as did vitamin D (36.17), thyroid stimulating hormone (TSH) (0.59), and creatinine of (244.1) with estimated glomerular filtration rate (eGFR) (19) (Table 1).

**Table 1:** Patient's initial laboratory investigation values PHT: parathyroid hormone, TSH: thyroid stimulating hormone

	Patient	Reference range
PHT	109.7	18.5-88 pg/ml
Ca	2.26	2.12-2.62 mmol/l
Vitamin D	36.17	20-80 ng/ml
TSH	0.59	0.35-5.5
Creatinine	244.1	61.9-114.9 umol/L

**Figure 1:**
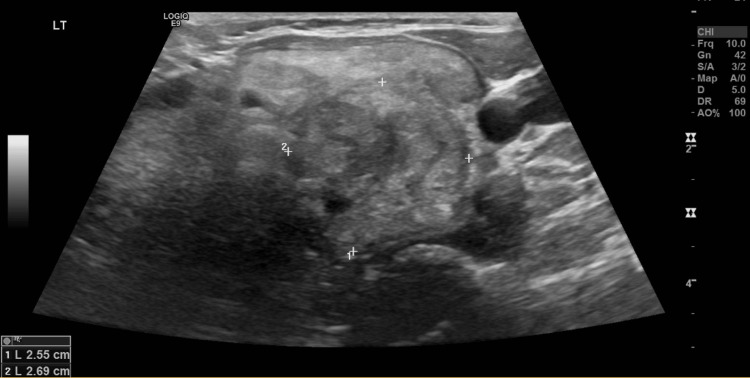
Left thyroid lobe ultrasonography showing a suspicious nodule

Neck sonography revealed diffuse enlargement of the thyroid gland harboring multiple left lobe nodules, with the most suspicious one being the largest measuring >2.5 cm, classified as TI-RADS 4 (moderately suspicious) (Figure 1). A fine-needle aspiration cytology (FNAC) of the thyroid revealed a Bethesda category 3 (atypia of undetermined significance).

**Figure 2:**
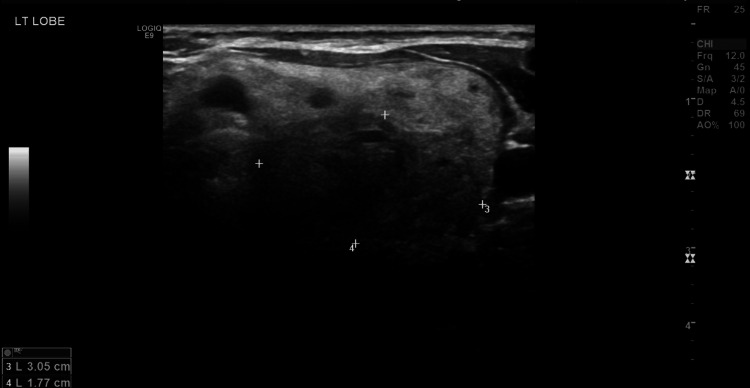
Follow-up ultrasound showing enlargement of the ill-defined left lobe nodule

**Figure 3:**
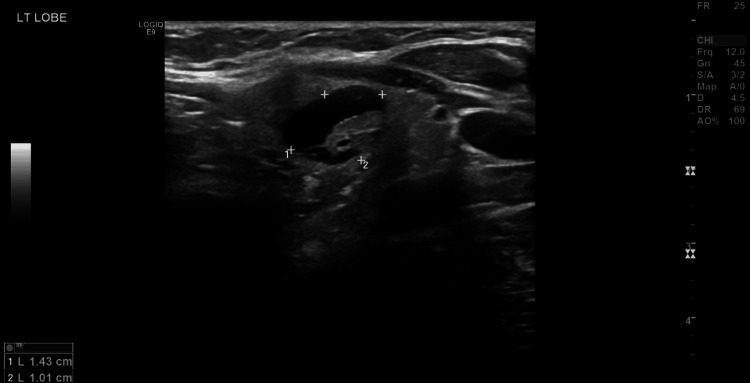
Left lobe hypoechoic nodule (lateral to the previous cystic nodule)

Initially, the patient was hesitant to opt for surgical intervention, so she was scheduled for follow-up. Interestingly, during the follow-up period, the patient had multiple visits to the orthopedic outpatient clinic for inflammatory tenosynovitis of the Achilles tendon and index finger. Sixteen months after the initial diagnosis, a neck ultrasound revealed an enlargement of a previously reported large, ill-defined left lobe nodule (3.0 x 1.7 cm) and another left lobe hypoechoic nodule that measured 14 x 10 mm. The right lobe showed enlargement with isoechoic nodules, the largest measuring 3.4 x 1.2 cm (Figures 2-3).

The patient underwent a total thyroidectomy. The perioperative and postoperative periods were uneventful. A histopathological evaluation of the sample was requested.

Gross examination showed a 64 x 48 x 28 mm right lobe, a 31 x 8 x 6 mm isthmus, and an 80 x 62 x 36 mm left lobe, collectively weighing 62 g. The cut surface was meaty, multinodular, and gelatinous, with a well-demarcated white-tan nodule in the left lobe.

**Figure 4:**
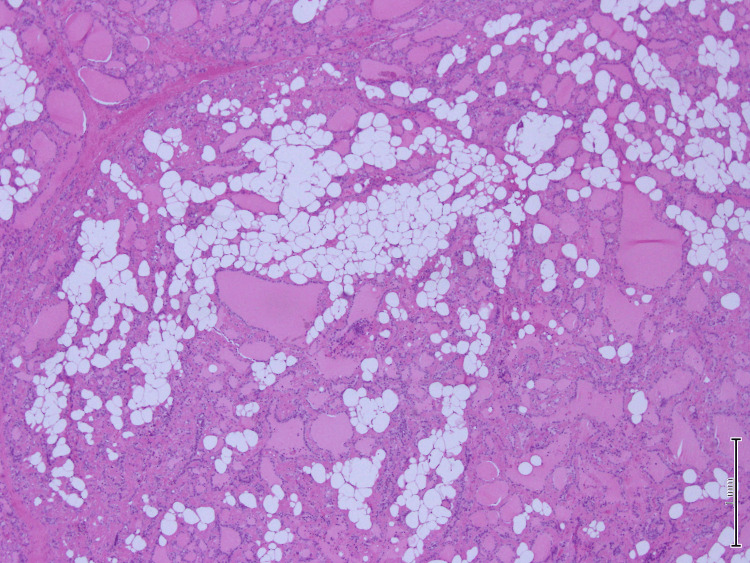
Hematoxylin-eosin stain x10: Intrathyroidal fatty metaplasia, slightly dilated follicles filled with colloid

**Figure 5:**
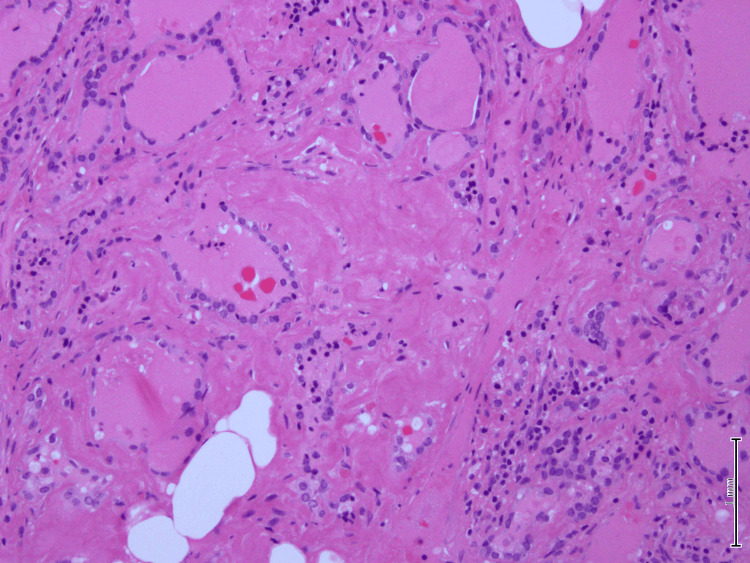
Hematoxylin-eosin stain x20: amorphous material deposits

**Figure 6:**
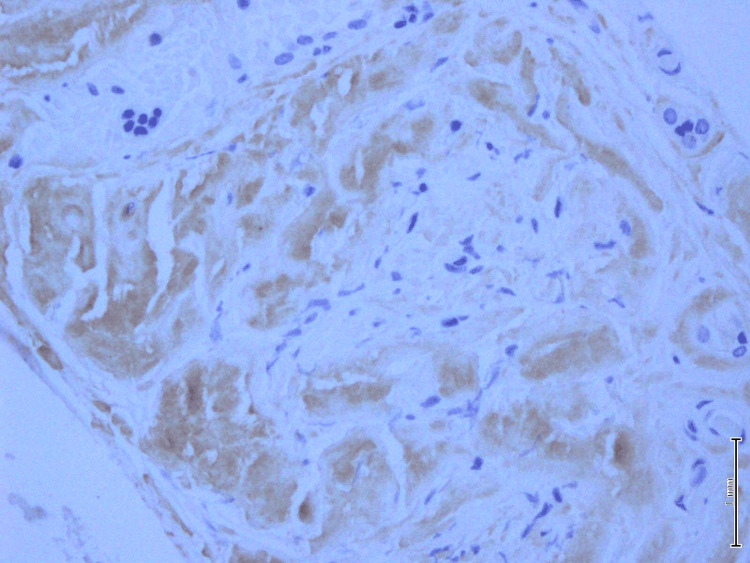
Amyloid-P IHC showing positivity

**Figure 7:**
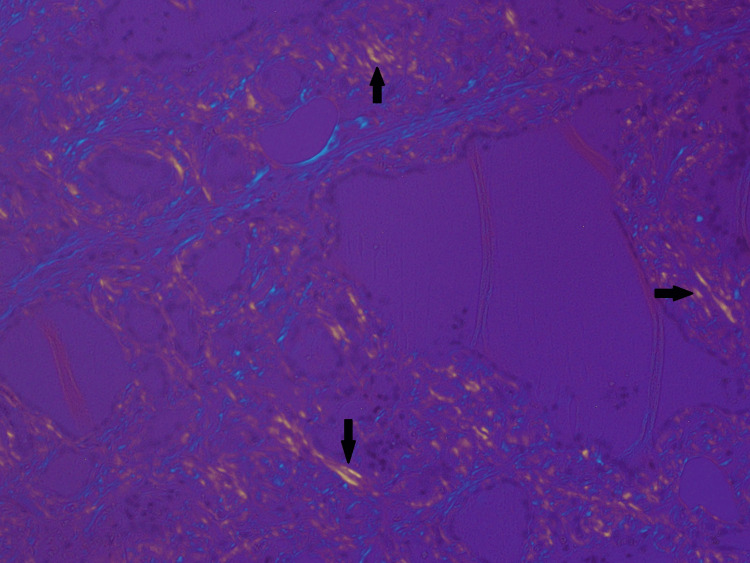
Congo red stain at 20x: showing apple-green birefringence (black arrows) under polarized microscopy

A microscopic examination of the left lobe lesion showed a follicular adenoma. The rest of the tissue shows vague nodules and marked deposition of dense amorphous material in the perifollicular and perivascular regions. There was extensive fatty metaplasia and a focal giant cell reaction. Immunohistochemistry for amyloid was positive. Congo red stain showed apple-green birefringence under polarized microscopy, which is consistent with amyloid deposition (Figures 4-7). 

The final diagnosis was consistent with AG. The patient was followed up in the outpatient clinic two weeks later with no active complaints.

## Discussion

Amyloidosis refers to the extracellular tissue deposition of fibrils composed of low-molecular-weight subunits of a variety of proteins [5]. It is well known that this deposition of proteins can affect many tissues, which explains the wide range of clinical manifestations of amyloidosis, which is affected mainly by the location, amount of proteins, and specific type of protein deposited. One of the classifications used for amyloidosis is primary amyloidosis (AL amyloidosis) and secondary amyloidosis (AA amyloidosis). It was actually found that in autopsy-based studies, intrathyroidal amyloid is present in approximately 80% of patients with secondary amyloidosis and in 50% of those with primary amyloidosis [1]. Dialysis-related amyloidosis (DRA) is one other type described in patients having dialysis for more than five years, and it is caused by deposits of beta-2 microglobulin that build up in the blood [5]. However, in our case, the patient, who is a known case of chronic kidney disease, was not on dialysis (eGFR was 19 at the time of diagnosis). Amyloid proteins tend to deposit in the thyroid gland in very few cases where the thyroid tissue can be infiltrated by amyloid material, causing thyroid gland enlargement [6-7]. This is not a common place for amyloid proteins to deposit, and as this condition is so rare and can only be confirmed by histopathological examination postoperatively, it is challenging for physicians to suspect it as the cause of goiter.

The first presentation of amyloidosis varies, as the protein can be deposited in any of the various tissues. In our case, AG seems to be the first confirmed manifestation of amyloidosis in this patient. This is not the first time to see such a finding, as previous case reports showed cases of such a rare presentation being the first presenting manifestation [8]. However, this finding leads to further thinking about the possibility that kidney failure in this patient may need more follow-up, as this may point to the possibility that amyloid deposition in the kidney may be already or ongoing, causing further worsening of kidney damage.

An FNA biopsy has been used to aid in the diagnosis of AG, mainly to exclude other differential diagnoses. It is a valuable, safe, and easily performed procedure. However, the definitive diagnosis of AG is most often made after thyroidectomy, as FNA is considered to have a limited role in the diagnosis of AG [2].

In conclusion, AG is a rare condition that is challenging for physicians to suspect, especially when the first presentation is in the thyroid gland. This is further complicated by the lack of concomitant chronic systemic inflammatory processes or a long history of dialysis. This case presented a patient with amyloidosis of the thyroid gland that was found on the histopathological examination after undergoing a thyroidectomy.

## Conclusions

Our patient is one of the few rare cases of AG in Bahrain. Findings of an enlarged thyroid gland have a wide differential diagnosis, and it is usually rare to suspect AG, especially as a first manifestation of amyloidosis. Histopathologic examination is the sole way of establishing a conclusive diagnosis. AG should be considered one of the rare differential diagnoses of an enlarged thyroid gland.
